# DECIDE: a cluster-randomized controlled trial to reduce unnecessary caesarean deliveries in Burkina Faso

**DOI:** 10.1186/s12916-019-1320-y

**Published:** 2019-05-02

**Authors:** Charles Kaboré, Valéry Ridde, Nils Chaillet, Fadima Yaya Bocoum, Ana Pilar Betrán, Alexandre Dumont

**Affiliations:** 1 IRD (French Institute for Research on sustainable Development), CEPED (IRD-Université Paris Descartes), Universités Paris Sorbonne Cités, ERL INSERM SAGESUD, Paris, France; 2 Research Institute of Health Sciences, Ouagadougou, Burkina Faso; 3 University of Montreal Public Health Research Institute (IRSPUM), Montreal, Canada; 4 Hospital Center of Laval University (CHUL), Quebec, Canada; 5 UNDP/UNFPA/UNICEF/WHO/World Bank Special Programme of Research, Development and Research Training in Human Reproduction (HRP), Department of Reproductive Health and Research, World Health Organization, Avenue Appia 20, CH-1211, Geneva 27, Switzerland

**Keywords:** Caesarean section, Mobile phone-based interventions, Clinical audit, Training

## Abstract

**Background:**

In Burkina Faso, facility-based caesarean delivery rates have markedly increased since the national subsidy policy for deliveries and emergency obstetric care was implemented in 2006. Effective and safe strategies are needed to prevent unnecessary caesarean deliveries.

**Methods:**

We conducted a cluster-randomized controlled trial of a multifaceted intervention at 22 referral hospitals in Burkina Faso. The evidence-based intervention was designed to promote the use of clinical algorithms for caesarean decision-making using in-site training, audits and feedback of caesarean indications and SMS reminders. The primary outcome was the change in the percentage of unnecessary caesarean deliveries. Unnecessary caesareans were defined on the basis of the literature review and expert consensus. Data were collected daily using a standardized questionnaire, in the same way at both the intervention and control hospitals. Caesareans were classified as necessary or unnecessary in the same way, in both arms of the trial using a standardized computer algorithm.

**Results:**

A total of 2138 and 2036 women who delivered by caesarean section were analysed in the pre and post-intervention periods, respectively. A significant reduction in the percentage of unnecessary caesarean deliveries was evident from the pre- to post-intervention period in the intervention group compared with the control group (18.96 to 6.56% and 18.27 to 23.30% in the intervention and control groups, respectively; odds ratio [OR] for incremental change over time, adjusted for hospital and patient characteristics, 0.22; 95% confidence interval [CI], 0.14 to 0.34; *P* < 0.001; adjusted risk difference, − 17.02%; 95% CI, − 19.20 to − 13.20%).

The intervention did not significantly affect the rate of maternal death (0.75 to 0.19% and 0.92 to 0.40% in the intervention and control groups, respectively; adjusted OR 0.32; 95% CI 0.04 to 2.23; *P* = 0.253) or intrapartum-related neonatal death (4.95 to 6.32% and 5.80 to 4.29% in the intervention and control groups, respectively, adjusted OR 1.73; 95% CI 0.82 to 3.66; *P* = 0.149). The overall perinatal mortality data were not available.

**Conclusion:**

Promotion and training on clinical algorithms for decision-making, audit and feedback and SMS reminders reduced unnecessary caesarean deliveries, compared with usual care in a low-resource setting.

**Trial registration:**

The DECIDE trial is registered on the Current Controlled Trials website: ISRCTN48510263.

**Electronic supplementary material:**

The online version of this article (10.1186/s12916-019-1320-y) contains supplementary material, which is available to authorized users.

## Introduction

Despite long-standing international concern and debate, the number of births by caesarean section continues to increase worldwide [1, 2]. The increase in the use of caesarean section is not limited to high-resource settings but affects low-income countries and their public hospitals [3]. In these settings, the increased use is likely to contribute to the worsening of maternal and perinatal outcomes [4, 5]. In addition, particularly in low- but also in middle-income countries, overuse and underuse of caesarean section co-exist, widening health inequalities and further weakening health systems in these countries [6]. With an extremely low national caesarean rate of approximately 2% [7], in Burkina Faso, local healthcare policies focus on increasing the caesarean delivery rate [8]. However, in some tertiary hospitals in Burkina Faso, the caesarean delivery rate may rise up to 40% with unclear medical justification [7]. Indeed, in this country, user fees for caesarean delivery were reduced by 80% since 2006 in all public hospitals [8], and totally eliminated in April 2016. The fee exemption includes hospital costs and transport costs for referred women. Cost was an important barrier to accessing caesarean sections prior to the fee subsidy policy, and addressing this barrier is necessary to ensure women who need a caesarean receive one; however, there are concerns that such policies may also increase unnecessary caesareans [9]. Unnecessary use of caesarean delivery increases the risk of maternal and perinatal morbidity [2, 10]. Therefore, the implementation of this policy should also include measures to prevent a rise in unnecessary caesarean deliveries.

Excessive caesarean rates in sub-Saharan African hospitals have been attributed to a lack of use and awareness of evidence-based clinical guidelines by healthcare professionals for appropriate caesarean decision-making [7, 11]. In Burkina Faso, the decision for caesarean birth may be made by general practitioners or midwives who are less trained and effective in obstetric decision-making than obstetricians-gynaecologists [12]. Medically unnecessary caesarean sections are associated not only with adverse health outcomes for mothers and newborns but also with high healthcare expenses for countries with already limited resources [10, 13].

Systematic reviews of strategies designed to increase compliance with evidence-based clinical guidelines among healthcare professionals provide evidence that two strategies are effective to reduce caesarean deliveries in settings with excessive rates: (i) mandatory second opinion and (ii) clinical audits, either alone or in combination with staff training, facilitation by a local opinion leader or supervision [14, 15]. A policy of mandatory second opinion is not feasible for settings with insufficient senior clinicians (obstetricians-gynaecologists) [16]. Audit and feedback have been used in various contexts, including low-income countries [14].

When supported with education or an opinion leader, audits of caesarean indications have resulted in small but significant reductions in the rates of caesarean deliveries in middle- and high-income countries [15]. Although some studies have shown that regular access to evidence-based health information via SMS or mobile-based decision-support systems may improve the adherence of healthcare professionals to management algorithms in low-income countries [17], to our knowledge, there are no studies assessing the value of conducting caesarean audits to reduce unnecessary caesarean births in these countries.

We designed a trial to assess the effect of a multifaceted intervention on reducing unnecessary caesarean sections in low-resource settings. The intervention aimed to increase the use of evidence-based algorithms for caesarean decision-making and included education and training on the algorithms, audits and timely feedback of caesarean sections as well as SMS reminders.

## Methods

### Hospitals and participants

We conducted the DECIDE trial (DECIsion for caesarean DElivery) at 22 public hospitals in Burkina Faso from May 2, 2014, to November 2, 2016. We included public hospitals with a functioning operating room, at least 200 caesarean sections performed in the year before the initiation of the study, no previously implemented audits of caesarean indications, and signed consent forms from the director of the hospital and the head of the maternity ward to participate. National academic hospitals were excluded because of the high number of junior clinicians in training (student midwives or doctors, interns or residents).

All healthcare professionals involved in caesarean decision-making in the participating hospitals were included in the study. These included doctors (general practitioners and obstetrician-gynaecologists) and midwives. The first 100 women and their newborns who delivered by caesarean section in each participating hospital during the pre- and the post-intervention periods were included in the analysis regardless of the reason or timing of the caesarean. Women whose caesareans were performed in another hospital and who were subsequently transferred to a participating hospital were not included in the study.

### Study design

The DECIDE trial was a stratified, facility-based parallel cluster-randomized trial. To avoid contamination bias between clinicians in the same service, the unit of randomization and intervention was the hospital, while the unit of analysis was the women who delivered by caesarean section. Randomization was stratified according to three different types of hospitals: regional hospitals, district hospitals in the two largest cities (Ouagadougou and Bobo Dioulasso) and district hospitals outside those two cities.

The study included a 6-month pre-intervention (baseline) period from May 2 to November 2, 2014, a 1-year intervention period from May 2, 2015, to April 30, 2016, and a 6-month post-intervention period from May 2 to November 2, 2016. After the baseline period, hospitals were randomly assigned to the intervention group or control group. All participating hospitals were randomly allocated simultaneously to minimize the risk of allocation bias. To avoid imbalance in the size of the two groups, we used computer-generated, blocked randomization within each stratum, with blocks consisting of four centres or, for strata with fewer than eight hospitals, two centres. Investigators were informed of the allocation just before the rollout of the intervention.

### Data collection

Information on the women who underwent caesarean sections during the study was abstracted by trained midwives from hospital registers and medical records, whose quality and archiving were regularly monitored by the study coordinator. Data were collected daily using a standardized questionnaire, in the same way at both the intervention and control hospitals. Data completeness and quality were assessed during daily maternities’ staff meetings, through quarterly on-site visits and queries sent to on-site data collectors to resolve discrepancies identified by the data manager. Data collectors were aware of the randomization assignments but were not involved in outcome assessments. Access to the database was restricted to the data manager until the trial was completed.

### Intervention and implementation

Evidence-based clinical algorithms were developed during the baseline period of the trial to help healthcare professionals in the caesarean decision-making process for the four main indications for caesarean reported by clinicians in Burkina Faso [18], namely, labour dystocia (obstructed/prolonged labour), foetal distress, previous caesarean section and pre-eclampsia/eclampsia, which represent 80.3% of all caesareans in study hospitals in the baseline period. We conducted a literature review to discern the diagnostic reasoning underlying evidence-based indications for caesareans (Additional file 1) and to generate a provisional list of good practice criteria, with preference given to evidence obtained through randomized controlled trials. The provisional list of criteria was sent to 16 international and national experts (gynaecologist-obstetricians, midwives and a public health physician) (Additional file 2), who gave their opinions on the relevance of each criterion and proposed others. The criteria retained were those validated by at least two thirds of the experts. We then developed clinical algorithms (for details, see Additional files 3, 4, 5, 6, and 7) for managing the four main indications for caesareans on the basis of these agreed criteria, as well as the corresponding definitions of unnecessary caesarean sections.

The first 3 months of the 1-year intervention period focused on the training of healthcare professionals. The chiefs of the maternity units of the hospitals in the intervention group were trained to use these clinical algorithms (2-day training) and to conduct clinical audits of caesarean indications (1-day training). Subsequently, these trained chiefs set up audit committees in their own hospitals (which consisted of physicians and midwives) and trained all healthcare professionals to use the algorithms for caesarean decision-making. Algorithms were printed on posters and posted in the delivery room of each hospital in the intervention group. With a view to sustainability, no financial incentive was provided to the chiefs or healthcare professionals. The initial provider training, conducted in Ouagadougou, the capital city of Burkina Faso, was led by two experts of the Society of Gynaecologists and Obstetricians of Burkina (SOGOB). The training was based on the WHO guidelines for managing complications of pregnancy and childbirth [19] and for clinical auditing [20].

During the 9 months after the training period, iterative weekly SMS reminders for appropriate caesarean decision-making were sent to all healthcare professionals involved in caesarean decision-making in intervention hospitals (Additional file 8), and audits of caesarean indications were launched by audit committees with the support of one researcher (CK) during his quarterly educational outreach visits. Monthly audits were recommended, and each audit cycle included five standardized steps according to the approach proposed by the WHO [20] [1]: identification of women who had caesarean deliveries for the main indications addressed by the clinical algorithms during the previous month [2]; data collection regarding the management of labour and delivery on standardized forms [3]; assessment by the local audit committee, with the use of clinical algorithms, of the relevance of the indications for caesarean delivery [4]; formulation of recommendations for best practices and the evaluation of previous recommendations, both performed by the committee; and [5] provision of informal and formal feedback to healthcare professionals. During the 6-month post-intervention period, healthcare professionals in the intervention group were encouraged to continue performing clinical audits without supervision, to assess sustainability.

No intervention was planned for the control group as part of this project. To assess contamination bias, we searched for any quality improvement programmes ongoing during the study period in the control hospitals that could impact caesarean rates. We also monitored staff turn-over and transfers between hospitals. We did not control or monitor if SMS reminders were shared or forwarded from staff in the intervention arm to staff in the control arm because this was not technically possible.

### Outcomes

The primary outcome was the percentage of unnecessary caesarean sections among all caesareans. Fifteen clinical categories of unnecessary caesareans were prespecified on the basis of the literature review and expert consensus (Table 1), grouped under the main four indications reported in the hospitals of Burkina Faso [18]. To avoid classification bias, caesarean sections were classified as necessary or unnecessary based on a standardized computer algorithm. This algorithm, developed as part of this study, was based on the established criteria (Table 1) and was applied to the database.

**Table 1:** Definition of unnecessary caesarean delivery

Foetal distress	Labour dystocia	Previous caesarean section	Pre-eclampsia/eclampsia
• Caesarean for foetal distress with clear amniotic fluid, normal^1^ temperature, normal progression of cervical dilation and foetal descent, and normal foetal heart rate (120–160 beats/min) • Caesarean for foetal distress with abnormal foetal heart rate (< 120 or > 160 beats/min or repeated deceleration) or coloured amniotic fluid without implementation of intrauterine resuscitative measures (oxygen administration and the mother in the left lateral decubitus position) • Caesarean for foetal distress at full cervical dilation with engaged head and without attempted instrumental delivery (forceps or vacuum)	• Caesarean for suspected foetal macrosomia without ultrasonography or clinical evidence of macrosomia • Caesarean for slow dilatation with intact membranes • Caesarean for slow dilation with ruptured membranes, adequate^2^ uterine contractions and arrest < 4 h • Caesarean for slow dilation with ruptured membranes, inadequate uterine contractions, and no augmentation with oxytocin • Caesarean for slow dilation with ruptured membranes, augmentation with oxytocin, and arrest < 6 h • Caesarean at full cervical dilation for non-engagement of presentation with women allowed to push for less than 2 h • Caesarean at full cervical dilation with engaged head and without attempted instrumental delivery (forceps or vacuum)	• Caesarean without contraindication for a trial of labour after a single caesarean section with a transversal scar, a singleton foetus in cephalic presentation, no ultrasonography or no clinical evidence of macrosomia, no pelvimetry or no clinical evidence of a restricted pelvis • Caesarean during labour without protraction or arrest disorders, lack of evidence of foetal distress, no documented signs of uterine rupture • Caesarean at full dilation with engaged head and without attempted instrumental delivery (forceps or vacuum)	• Caesarean for pre-eclampsia with lack of evidence of foetal distress^3^ or foetal growth restriction^4^, lack of signs of severity for the woman^5^ and normal progression of cervical dilation (> 2 cm in 4 h) • Caesarean for pre-eclampsia or eclampsia with normal progression at full dilation (no arrest > 3 h)

^1^Temperature of 37.5 °C

^2^Three contractions in 10 min, each contraction lasting more than 40 s

^3^Fetal heart rate (between 120 and 160 beats/min) without slowing

^4^Weight below the 10th percentile for gestational age

^5^Signs of severe pre-eclampsia: blood pressure ≥ 160/110 mmHg; albuminuria, with urinary albumin ≥ 3+ or ≥ 3 g/24 h; oliguria, with urinary flow < 30 mL/h; headache; epigastric pain; vision disorders; neurological disorders; seizures; haemolysis; low platelet count and high levels of liver enzymes

Secondary outcomes included the percentage of unnecessary caesareans for each of the four indications; the relative contribution of each indication and each group of the Robson classification [3] to all caesarean sections performed; the percentage of caesarean sections performed before and after the onset of labour; the rates of intra-hospital maternal death among women delivering by caesarean section; intrapartum-related neonatal death (fresh stillbirths and immediate neonatal deaths before 24 h) among births by caesarean section; and quality caesarean decision-making score among healthcare professionals. Clinical decision-making competency and skills were evaluated using hypothetical patient vignettes framed around selected decisional algorithms (33 vignettes and 51 related questions) [12]. The results of this evaluation were used to generate a decision-making score for the main indications of caesarean section in Burkina Faso.

During quarterly visits, CK conducted participant observations of audit committee meetings in intervention hospitals. The healthcare professionals’ views on the relevance of the caesarean indications were collected, as well as on the reasons for unnecessary caesarean sections and their recommendations for action.

### Sample size

The sample size was calculated to maximize statistical power while minimizing the number of clusters [21]. To account for clustering by hospital, we assumed an intraclass correlation coefficient of 0.02, estimated based on the percentage of unnecessary caesareans in 10 hospitals in Burkina Faso [7]. We calculated that we would have to enrol 22 hospitals, with a total of 2200 women delivering by caesarean section each in the baseline and post-intervention period, for the study to have 80% power to detect a 50% relative reduction with the intervention in the percentage of unnecessary caesareans, assuming a baseline percentage of 25%, at a two-sided alpha significance level of 0.05.

### Statistical analysis

In the primary intention-to-treat analyses, the intervention effect on the primary outcome was estimated as the difference between the allocation groups in the change in individual women’s risk of unnecessary caesarean birth from the baseline to the post-intervention period. The binary individual-level outcome relied on the generalized estimating equation (GEE) extension of the logistic regression model, with an exchangeable covariance structure, to account for the clustering of women within hospitals [22]. Using the difference-in-differences approach [23], the additional reduction in the percentage of unnecessary caesareans in the intervention group, relative to the reduction in the control group, was estimated by the odds ratio (OR) with the 95% confidence interval (CI) for the interaction between indicators of the trial group (intervention vs control) and time (post-intervention vs baseline) from the GEE model. The GEE model-based two-sided Wald test of this interaction, at *α* = 0.05, was used to test the significance of the intervention effect. The same approach was used to assess the effect of the intervention on hospital-based maternal and intrapartum-related neonatal mortality. In the sensitivity analysis, for the primary outcome, we considered all caesarean deliveries for previous caesarean sections as non-avoidable caesareans because women may request an elective caesarean section to prevent maternal or perinatal poor outcomes.

The GEE model for the primary outcome was adjusted for the stratification variable, namely, hospital type, as well as for variables selected a priori as potential risk factors for unnecessary caesareans [18], including (a) the baseline characteristics of the hospitals (systematic use of a partograph, the 24-h availability of laboratory tests and the 24-h availability of an anaesthetist), (b) the qualifications of the healthcare professional who decided on the caesarean section (qualifications) and (c) the characteristics of the individual women (spouse’s and woman’s occupations, spouse’s and woman’s education levels, lack of a prenatal visit, maternal age, referral from another healthcare facility and time of caesarean section). To assess whether the intervention effect varied according to hospital type, we tested the corresponding three-way interactions: hospital type × intervention × time at two-tailed *α* = 0.05. All secondary binary outcomes related to caesarean practice and case fatality were analysed using the same methods as those for the primary outcome. The GEE model was then adjusted for variables selected a priori as potential risk factors for intra-hospital maternal mortality, including (a) hospital baseline characteristics (24-h availability of laboratory tests and an anaesthetist), (b) qualification of the healthcare professional who performed the caesarean, and (c) women’s characteristics (residence, age, parity, previous caesarean delivery, any pathology during pregnancy, prenatal visit attendance, multiple pregnancy, referral from another health facility, caesarean performed before labour vs during labour). To assess the effect of the intervention on perinatal mortality, the model was adjusted for the same variables used for maternal mortality plus birth weight. To assess the effect of the intervention on clinical decision-making for caesarean section, quantified by the quality decision-making score, we adapted the difference-in-differences approach, described above for the primary outcome, to the analysis of a quantitative healthcare professional-level outcome. Specifically, for each score, we estimated the multivariable mixed linear model, with 218 healthcare professionals (123 at the pre-intervention period and 95 at the post-intervention period) as the units of the analysis. An exchangeable covariance structure was assumed to account for the correlation between the two complexity scores, namely, the baseline period and post-intervention period, within the same hospital. The multivariable mixed linear model was adjusted for the effects of the year, the randomization group and their interaction for the stratification variable. Statistical analyses were conducted by one of the co-authors (NC) who was unaware of the hospital assignments. All analyses were conducted using Stata version 12.0. as well as SAS, version 9.3, to check the accuracy. This study is registered with Current Controlled Trials, as number ISRCTN48510263.

### Additional non-prespecified analyses

The WHO Statement proposes the use of the Robson classification as the global standard for assessing, monitoring and comparing caesarean rates within healthcare facilities over time, and between facilities [2].

In this paper, we report the contribution of each group of the Robson classification as a secondary outcome which was not stated in the protocol. Indeed, the 2015 WHO recommendation to use this classification was made after the writing of the protocol.

## Results

**Fig. 1:**
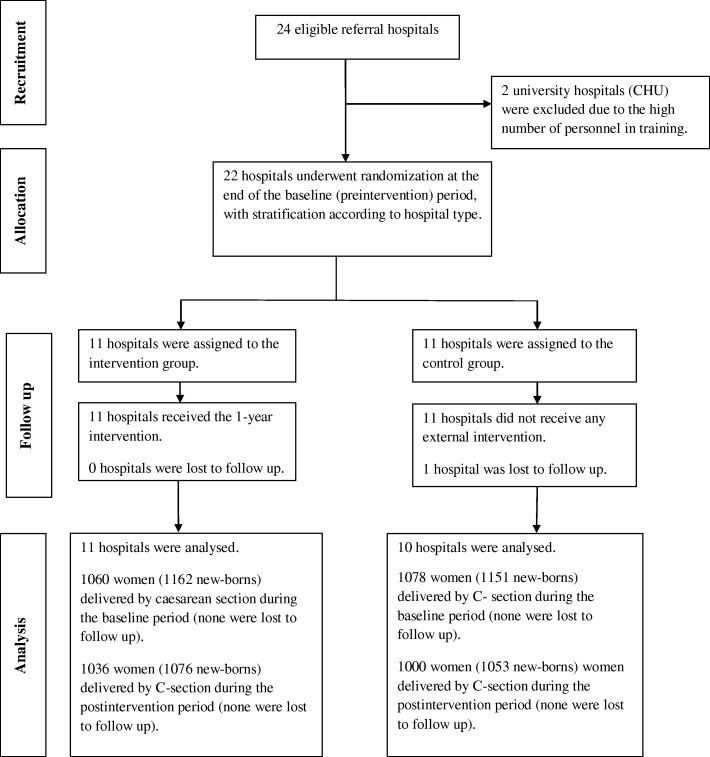
Flow chart showing records included in this study as well as reasons for exclusion

All 22 hospitals meeting the eligibility criteria agreed to participate in the trial (five district hospitals in the two main cities, eight district hospital outside these main cities and nine regional hospitals). One hospital was lost to follow-up because the operating room was not functional during the post-intervention period (Fig. 1). The overall caesarean section rate changed from 21.4% (baseline) to 20.0% (post-intervention) in the hospitals in the intervention group and from 18.7% (baseline) to 20.3% (post-intervention) in the control hospitals (absolute risk difference − 3.1%; 95% CI − 7.2 to 2.6%, *P* = 0.203). The 4174 women who delivered by caesarean were included in chronological order of admission for analysis: 2138 during baseline period and 2036 during post-intervention period. The number of included women varied from 60 to 100 within participating hospitals during the pre-intervention period and from 54 to 100 during post-intervention period. Three hospitals during the pre-intervention period and two hospitals during the post-intervention period did not reach the expected number of 100 caesareans. Among the included women, 3290 (79%) had caesarean deliveries for the four selected indications, and none of the included women were lost to follow-up.

The baseline characteristics of the women were very similar between groups, except for hospital type (due to the limited number of facilities randomized). The rate of caesareans performed in a regional hospital was higher in the intervention group than in the control group (Table 2).

**Table 2:** Baseline characteristics of the hospitals (*n* = 22), patients (*n* = 2138) and newborns (*n* = 2313)

	Intervention		Control	
	Hospitals (*n* = 11)	Patients (*n* = 1060)	Hospitals (*n* = 11)	Patients (*n* = 1078)
Hospital characteristics				
Type of hospital				
Regional hospitals	5	500 (47.16)	4	400 (37.10)
District hospitals in main cities	2	200 (18.86)	3	300 (27.82)
Other district hospitals	4	360 (33.96)	4	378 (35.06)
Healthcare professionals per hospital, mean (SD)				
Obstetrician-gynaecologist	1.7 (2.8)		1.6 (2.8)	
General practitioner	0.7 (0.6)		0.4 (0.5)	
Midwife	19.3 (6.4)		20.3 (7.6)	
Patient characteristics				
Maternal age > 35 years		107 (10.09)		108 (10.01)
Nulliparous		397 (37.45)		403 (37.38)
Previous caesarean section		249 (23.49)		214 (19.85)
No prenatal visit		412 (38.86)		383 (35.52)
Any pathology during current pregnancy		241 (22.73)		245 (22.72)
Multiple pregnancy		68 (6.41)		61 (5.65)
Presentation of infant				
Cephalic		930 (87.73)		963 (89.33)
Breech		76 (7.16)		77 (7.14)
Transverse		54 (5.09)		38 (3.50)
Referral during labour		715 (67.45)		760 (70.50)
Induced labour		11 (1.03)		14 (1.29)
Caesarean indication				
Foetal distress		342 (32.16)		328 (30.42)
Prolonged or obstructed labour		252 (23.77)		256 (23.74)
Previous caesarean section		187 (17.64)		191 (17.71)
Pre-eclampsia		71 (6.69)		62 (5.75)
Other indication		208 (19.62)		241 (22.35)
Caesarean decision-maker				
Obstetrician-gynaecologist		541 (51.03)		550 (51.02)
General practitioner		290 (27.35)		295 (27.36)
Midwife		229 (21.60)		233 (21.61)
Time of caesarean indication				
Planned before labour		181 (17.07)		173 (16.04)
Emergency intrapartum		879 (82.93)		905 (83.95)
Neonatal birth weight				
< 1500 g		17 (1.46)		19 (1.65)
1500–2499 g		152 (13.08)		182 (15.81)
2500–3999 g		923 (79.43)		913 (79.32)
≥ 4000 g		62 (5.33)		45 (3.90)

Values are the number with the indicated characteristic/number in the group (percentage) unless stated otherwise

The percentage of unnecessary caesareans in the pre-intervention period was similar in the two groups (18.9% and 18.2% in the intervention and control groups, respectively), and the post-intervention rate increased to 23.3% in the control group but decreased to 6.5% in the intervention group (Table 3). From the pre-intervention period to the post-intervention period, there was a significant reduction in the percentage of unnecessary caesareans in the intervention group compared with that in the control group, with an adjusted OR of 0.22 (CI, 0.14 to 0.34; *P* < 0.001) and an adjusted absolute risk difference of − 17.02 (95% CI, − 19.20 to − 13.20) (Table 3). The sensitivity analysis in which all caesarean deliveries for previous caesarean section were considered non-avoidable found a very similar intervention effect (adjusted OR 0.22; 95% CI 0.12 to 0.38). The intervention effect did not vary significantly across hospitals with different levels of care (*P* = 0.92 for the three-way interaction).

**Table 3:** Unnecessary caesarean delivery by group allocation and period (see Table 1 for the definition of the 15 indications of unnecessary caesareans)

Factor	Intervention group			Control group			Crude difference in rate change (95% CI)	Intervention effect^a^		
	Baseline (*n* = 1060)	Post-intervention (*n* = 1036)	Diff.	Baseline (*n* = 1078)	Post-intervention (*n* = 1000)	Diff.		Adjusted absolute risk difference (95% CI)	Adjusted odds ratio (95% CI)	*P* value
Unnecessary caesarean	18.96 (201/1060)	6.56 (68/1036)	− 12.40	18.27 (197/1078)	23.30 (233/1000)	5.03	− 12.80 (− 16.61 to − 9.08)	− 17.02 (− 19.20 to − 13.20)	0.22 (0.14 to 0.34)	0.000
Foetal distress	28.1 (96/342)	5.1 (11/215)	− 22.96	30.5 (100/328)	46.9 (108/230)	16.4	− 39.4 (− 45.6 to − 7.7)	− 39.6 (− 44.4 to − 30.0)	0.09 (0.03 to 0.23)	0.000
Labour dystocia	21.8 (55/252)	11.6 (49/420)	− 10.16	20.3 (52/256)	32.0 (113/353)	11.7	− 21.8 (− 22.4 to − 4.·7)	− 17·2 (− 23.4 to − 8.0)	0.37 (0.20 to 0.67)	0.001
Previous caesarean	18.0 (40/222)	5.4 (08/147)	− 12.57	18.5 (29/156)	7.1 (12/155)	− 11.4	− 1.2 (− 3.4 to 0.40)	− 3.9 (− 6.9 to 6.9)	0.47 (0.10 to 2.05)	0.316
Pre-eclampsia	14.1 (10/71)	0 (0/31)	− 14.07	25.8 (16/62)	0 (0/50)	− 25.8	–	–	–	–

Data are the numbers of women per 100 caesarean sections (percentages of unnecessary caesarean section among all caesareans performed) by group allocation and period

^a^Estimation of the intervention effect is adjusted for the place of residence, spouse’s occupation, woman’s occupation, patient’s education level, spouse’s education level, antenatal care attendance, maternal age, referral from another healthcare facility, time of caesarean indication, hospital type, availability of partograph, qualifications of caesarean decision-maker, 24-h on-site presence of staff performing anaesthesia and 24-h presence on-site of laboratory tests

Unnecessary caesareans were significantly reduced among caesareans performed for foetal distress (adjusted risk difference, − 39.6%; 95% CI, − 44.4 to − 30.0; *P* < 0.001) and labour dystocia (adjusted risk difference, − 17.2%; 95% CI, − 23.4 to − 0.8; *P* = 0.001). The intervention had no effect on reducing the percentage of unnecessary caesareans related to repeat caesarean (Table 3). We could not assess the effect on caesareans performed for pre-eclampsia/eclampsia because none of the caesareans for this indication were classified as unnecessary in the post-intervention period in either group.

Table 4 presents the distribution of caesareans performed during the study by the Robson group, indication and maternal and neonatal case fatality. Women without a previous uterine scar, with a singleton cephalic pregnancy, at term in spontaneous labour represented Group 1 (primiparous) and Group 3 (multiparous) of the Robson classification [3]. These groups were the two largest contributors to the overall caesarean section rates at baseline: 25.47% for Group 1 and 24.24% for Group 3 in the intervention hospitals, and 23.46% for Group 1 and 24.11% for Group 3 in the control hospitals. After adjusting for maternal and hospital characteristics, the relative contribution of each group to the overall caesarean section rate was significantly reduced in the intervention hospitals, compared with the control hospitals. This effect was comparable between primiparous (adjusted OR of 0.66; 95% CI 0.47 to 0.94, *P* = 0.024) and multiparous women (adjusted OR of 0.70, 95% CI, 0.52 to 0.95, *P* = 0.022). The intervention resulted in a marginally significant decrease in the relative contribution of women with multiple pregnancies (Group 8) to the overall caesarean section rate (adjusted OR of 0.63; 95% CI 0.40 to 1.00; *P* = 0.051). There was no significant effect of the intervention on the timing of caesarean section (before or after the onset of labour) or on the relative contribution of each indication to the overall caesarean section rate. The intervention did not significantly affect the rate of maternal death (0.75 to 0.19% and 0.92 to 0.40% in the intervention and control groups, respectively; adjusted OR 0.32; 95% CI 0.04 to 2.23; *P* = 0.253) or intrapartum-related neonatal death (4.95 to 6.32% and 5.80 to 4.29% in the intervention and control groups, respectively; adjusted OR 1.73; 95% CI 0.82 to 3.66; *P* = 0.149). The non-significance of the trends found does not exclude the possibility of differences as the trial was not powered for these outcomes (Table 4). The intervention group had a significant increase in the overall health professionals’ decision-making score compared with the control group (difference between mean changes, 3.42; 95% CI, 1.95 to 4.89; *P* < 0.001). The increase in the quality decision-making score was driven mostly by the effect of the intervention on heath professionals’ performance regarding caesarean indications for foetal distress and labour dystocia (Table 5).

**Table 4:** Selected characteristics of the caesarean births performed during the study birth period by group allocation and period

Factor	Intervention group			Control group			Crude difference in rate change (95% CI)	Intervention effect		
	Baseline (*n* = 1060)	Post-intervention (*n* = 1036)	Diff.	Baseline (*n* = 1078)	Post-intervention (*n* = 1000)	Diff.		Adjusted absolute risk difference (95% CI)	Adjusted odds ratio (95% CI)	*P* value
Contribution of each group to the overall caesarean section rate^a^ (*n*, %)										
Group 1	25.47 (270/1060)	25.09 (260/1036)	− 0.38	23.46 (253/1078)	23.90 (239/1000)	0.44	− 4.44 (− 9.60 to 0.75)	− 3.50 (− 5.70 to − 0.60)	0.66 (0.47 to 0.94)	0.024
Group 2	3.96 (42/1060)	3.47 (36/1036)	− 0.49	4.91 (53/1078)	2.30 (23/1000)	− 2.61	1.52 (− 0.73 to 3.77)	1.00 (− 0.40 to 3.40)	1.45 (0.82 to 2.56)	0.189
Group 3	24.24 (257/1060)	23.74 (246/1036)	− 0.50	24.11 (260/1078)	29.00 (290/1000)	4.89	− 6.56 (− 11.81 to − 1.31)	− 6.80 (− 11.5 to − 1.00)	0.70 (0.52 to 0.95)	0.022
Group 4	4.24 (45/1060)	3.47 (36/1036)	− 0.70	6.02 (65/1078)	4.40 (44/1000)	− 1.62	0.05 (− 2.45 to 2.56)	−0.10 (− 1.90 to 3.10)	0.98 (0.55 to 1.76)	0.969
Group 5	18.67 (198/1060)	18.24 (189/1036)	− 0.43	17.53 (189/1078)	16.40 (164/1000)	− 1.13	− 1.91 (− 6.53 to 2.70)	− 6.00 (− 6.00 to 6.90)	0.96 (0.59 to 1.55)	0.888
Group 6	1.79 (19/1060)	2.99 (31/1036)	1.20	2.41 (26/1078)	2.20 (22/1000)	− 0.21	0.96 (− 0.85 to 2.78)	1.20 (− 0.60 to 4.70)	1.57 (0.74 to 3.31)	0.236
Group 7	2.64 (28/1060)	4.72 (49/1036)	2.08	2.87 (31/1078)	2.90 (29/1000)	0.03	1.34 (− 0.80 to 3.49)	1.6 (− 0.30 to 4.90)	1.59 (0.89 to 2.85)	0.116
Group 8	6.13 (65/1060)	3.66 (38/1036)	− 2.47	5.93 (64/1078)	4.60 (46/1000)	− 1.33	− 2.06 (− 4.70 to 0.57)	− 1.70 (− 2.70 to 0.00)	0.63 (0.40 to 1.00)	0.051
Group 9	4.15 (44/1060)	5.88 (61/1036)	1.73	2.87 (31/1078)	4.50 (45/1000)	1.63	− 0.66 (− 3.13 to 1.80)	− 1.60 (− 2.80 to 1.30)	0.63 (0.36 to 1.30)	0.256
Group 10	8.67 (92/1060)	8.68 (90/1036)	0.01	9.83 (106/1078)	9.80 (98/1000)	− 0.03	− 1.35 (− 4.85 to 2.14)	− 1.00 (− 4.10 to 3.40)	0.89 (0.56 to 1.40)	0.619
Caesarean performed before labour (vs during labour)	17.07 (181/1060)	15.34 (159/1036)	− 1.73	16.04 (173/1078)	16.60 (166/1000)	0.56	− 1.16 (− 13.02 to 10.70)	− 1.14 (− 10.60 to 16.80)	0.90 (0.32 to 2.52)	0.846
Caesarean by indication (*n*, %)										
Foetal distress	32.26 (342/1060)	20.75 (275/1036)	− 11.41	30.42 (328/1078)	23.00 (230/1000)	− 7.42	− 0.41 (− 5.69 to 4.87)	−2.40 (−8.50 to 5.30)	0.87 (0.57 to 1.32)	0.518
Labour dystocia	23.77 (252/1060)	40.54 (420/1036)	16.77	23.74 (256/1078)	35.30 (353/1000)	11.56	4.59 (− 1.34 to 10.53)	4.10 (−4.90 to 13.80)	1.19 (0.80 to 1.77)	0.379
Previous caesarean	17.64 (187/1060)	14.18 (147/1036)	− 3.46	17.71 (191/1078)	15.50 (155/1000)	− 2.21	− 7.36 (− 11.79 to − 2.93)	−5.00 (− 100.10 to 3.90)	0.64 (0.31 to 1.31)	0.227
Pre-eclampsia/eclampsia	6.69 (71/1060)	2.99 (31/1036)	− 3.70	5.75 (62/1078)	5.00 (50/1000)	− 0.75	− 1.35 (− 3.96 to 1.25)	−1.80 (− 3.20 to 0.80)	0.63 (0.34 to 1.16)	0.143
Other indication	5.37 (57/1060)	8.30 (86/1036)	2.93	6.21 (67/1078)	8.10 (81/1000)	1.89	1.60 (− 3.40 to 6.62)	0.70 (− 2.00 to 4.50)	1.10 (0.74 to 1.63)	0.617
Case fatality (*n*, %)										
Maternal death	0.75 (8/1060)	0.19 (2/1036)	− 0.56	0.92 (10/1078)	0.40 (4/1000)	− 0.52	− 0.02 (− 0.91 to 0.87)	− 0.30 (− 0.40 to 0.50)	0.32 (0.04 to 2.23)	0.253
Intrapartum-related neonatal death	4.95 (53/1069)	6.32 (64/1012)	1.37	5.80 (63/1085)	4.29 (43/1002)	− 1.51	2.78 (− 0.18 to 5.76)	2.90 (− 0.70 to 9.80)	1.73 (0.82 to 3.66)	0.149

Data are the numbers of caesareans per 100 patients (percentages of unnecessary caesarean section) by group allocation and period

^a^The Robson ten-group classification system [3]

**Table 5:** Quality decision-making score among healthcare professionals by group allocation and period

Factor	Intervention group			Control group			Difference between^a^ Mean changes (95% CI)	*P* value
	Baseline (*n* = 76)	Post intervention (*n* = 53)	Diff.	Baseline (*n* = 47)	Post intervention (*n* = 42)	Diff.		
Overall score	33.97 (4.09)	39.30 (3.61)	5.33	33.82 (4.68)	35.90 (3.22)	2.08	3.42 (1.95 to 4.89)	0.000
Score for foetal distress	4.51 (1.50)	6.33 (0.89)	1.82	4.70 (1.48)	5.04 (1.14)	0.34	1.46 (0.58 to 2.34)	0.001
Score for labour dystocia	8.03 (1.36)	8.20 (0.86)	0.17	8.08 (1.99)	6.23 (1.26)	− 1.85	1.97 (1.21 to 2.73)	0.000
Score for previous caesarean	4.30 (0.98)	4.58 (0.81)	0.28	4.31 (1.02)	4.38 (0.79)	0.07	0.17 (− 0.15 to 0.51)	0.296
Score for pre-eclampsia/eclampsia	4.73 (0.91)	4.75 (0.91)	0.02	4.70 (0.83)	4.66 (0.78)	− 0.04	0.13 (− 0.24 to 0.52)	0.478
Score for other indications	12.38 (2.22)	15.32 (2.43)	2.94	12.02 (2.93)	15.59 (2.06)	3.57	− 0.63 (− 1.90 to 0.63)	0.327

Data are the mean score (standard deviation). Maximum score: 7 for foetal distress; 11 for labour dystocia; 6 for previous caesarean delivery; 6 for pre-eclampsia/eclampsia; 21 for other indications including premature rupture of membranes, intrauterine growth restriction, amniotic fluid anomalies, HIV seropositivity, post-term pregnancy, abruptio placentae and sequelae of genital mutilation

^a^Estimation of intervention effect adjusted for the type of hospital, systematic review of partograms and healthcare professionals^’^ qualifications and experience

The algorithms were posted in the delivery rooms of most intervention hospitals, except in two hospitals due to a lack of space. For optimal exposition, we decided to disseminate a guide containing the algorithms to each healthcare professional in the intervention hospitals. There were between three and four training sessions, with 10 to 15 participants, in participating hospitals. These training sessions were focused on clinical algorithms and lasted a half day each on average. The audit cycles were launched with respect to the approach proposed by the WHO [20]. The frequency of the audits varied depending on the number of caesareans conducted in each intervention hospital, and all caesareans performed for the indications addressed by the algorithms were audited. Indications related to foetal distress and labour dystocia were mostly unnecessary. There were between three and eight audit cycles by hospital during the intervention period. Between 11 and 23 healthcare professionals per hospital (median 16; interquartile range 11–21) attended the audit meetings where the results of the audits and recommendations were presented.

No other programmes that were specifically designed to reduce unnecessary caesarean deliveries were found to have been implemented in participating hospitals during the trial and only one midwife left an intervention hospital to move to a control hospital during the study period.

## Discussion

This multifaceted intervention, which was targeted to healthcare professionals and included availability and training on evidence-based algorithms for caesarean decision-making, audits of caesarean indications with timely feedback to healthcare professionals and SMS reminders, resulted in a significant reduction in unnecessary caesareans sections and improved the quality of decision-making for caesarean delivery. The intervention was also associated with a significant decrease in the relative contributions of Robson Groups 1 and 3 (women with a single cephalic pregnancy at term in spontaneous labour without a previous caesarean section) to the overall caesarean section rate. The intervention did not significantly affect the rates of maternal or intrapartum neonatal mortality among caesarean births.

These results may reflect improvements in the caesarean decision-making process in intervention hospitals. Indeed, all of the intervention hospitals planned regular meetings for caesarean indication audits with feedback to healthcare professionals and provided regular on-site training accordingly. SMS reminders and on-site training facilitated caesarean indication audits by providing healthcare professionals with the knowledge and confidence to make relevant suggestions to improve the quality of caesarean decision-making, particularly for clinical situations such as labour dystocia and foetal distress which were the main indications for caesarean section in this context [7, 11]. This is consistent with the improvement found in quality decision-making scores among health professionals.

There was a non-significant reduction in the overall caesarean rate in the intervention hospitals as compared with control hospitals. A change in policy between the pre- and post-intervention period further reduced the caesarean section fee from 20% to full exemption. The new policy with no charge for caesarean section may explain why the overall hospital caesarean rate in the intervention group was not significantly reduced [24]. The factors related to unnecessary caesareans were clearly identified, including clinical and organizational issues (e.g. lack of assessment of the indication by doctors before the caesarean section, lack of equipment for intrauterine resuscitation and lack of skills for the use of forceps or vacuum) which led to actionable solutions allowing the reductions of unnecessary caesareans. These factors are in accordance with those identified in previous studies in low-resource settings [10, 25, 26]. The audit and feedback system minimized these factors, by providing opportunities for personal development, enabling recognition from peers and women, fostering a participatory approach, and creating commitment to a shared aim (reduction in unnecessary caesareans).

The intervention had no effect on either the quality of decision-making for repeat caesarean delivery or on the relative contribution of the women with a previous uterine scar (Robson Group 5) to the overall caesarean section rate. As in other low-income countries, the caesarean rate among women with a previous uterine scar is high in Burkina Faso (more than 60%) [3], and oxytocin augmentation during labour in this clinical situation is not allowed in this country. Healthcare professionals are reluctant to implement a trial of labour after caesarean because of the lack of electronic foetal monitoring and the perception of a high risk of uterine rupture [27].

Insufficient qualifications and skills of healthcare professionals have been reported to be a main factor contributing to the increase in caesarean sections without a medical indication or unnecessary caesarean sections in low-resource settings [7, 18, 28]. The DECIDE intervention resulted in improved performance of healthcare professionals in the caesarean decision-making process, as shown by the increase in the quality decision-making score among staff with various qualifications. In the context of the unprecedented global increase in caesarean birth rates, the WHO emphasizes that rather than striving to achieve any specific rate, efforts should focus on providing caesareans to all women in need [2]. Strategies such as the one implemented in this trial go beyond reducing unnecessary caesareans to ensuring the appropriateness of the decision and improving the quality of the care and performance of the healthcare professional, which is critical in these low-resource settings regardless of the caesarean rate.

To our knowledge, this is the first trial in a low-income country to confirm the benefits of implementing evidence-based clinical guidelines to improve caesarean practice within a multifaceted intervention. Among 11 prior studies—randomized and nonrandomized in middle- and high-income countries—that assessed the effectiveness of a mandatory second opinion, audit and feedback or peer reviews, four showed significant reductions in the rates of caesarean delivery [15]. Among five randomized trials, only two showed a significant, albeit small, reduction (adjusted risk difference, − 1.9% and − 1.8%) [15].

These studies examined impact on the overall caesarean section rate, but not the quality of decision-making. There is a growing literature on unnecessary caesareans, but no consensus on a precise definition—a necessary step in order to design interventions that successfully reduce them. This lack of definition is understandable since there is no standard accepted algorithm that determines when a caesarean is necessary. Our definition is a first attempt to define this indicator; other studies might be interested in defining it in other contexts.

The DECIDE trial was conducted in a large and representative sample of referral hospitals in Burkina Faso. The safe reduction in the percentage of unnecessary caesareans observed in this trial and the moderate efforts required to maintain the programme (approximately 3 days per month to conduct an audit session, develop recommendations, provide feedback and review the implementation of the recommendations, and a half day to conduct in-site training) and the modest financial resources required (less than 0.4% of the annual budget of intervention hospitals) suggest that a similar intervention may be beneficial in other similar countries or regions struggling with unnecessary caesareans. Our study had some limitations. First, we audited only four main indications (foetal distress, pre-eclampsia/eclampsia, labour dystocia and previous caesarean). Therefore, the percentage of unnecessary caesareans among all caesarean cases is likely underestimated. Second, the rates of maternal and perinatal mortality were assessed among caesarean births only. The effect of the intervention on mortality may be different when considering all deliveries together (vaginal and caesarean births). The lack of outcome data among vaginal deliveries prevents ruling out a possible shift of intrapartum-related mortality from caesarean to vaginal births due to clinical practice change. However, given the in-service evidence-based education and training provided as part of the intervention, which was based on the WHO guidelines for managing complications of pregnancy and childbirth, this shift of mortality towards vaginal delivery is improbable. Third, the healthcare professionals in the intervention hospitals may have better documented the indications. But the observed effect of the intervention on unnecessary caesarean deliveries is likely the results of a real change in decision-making for caesarean delivery as shown by the results of Robson’s classification, the significant increase in decision-making score and the decrease in the overall caesarean section rate in the intervention group. Fourth, we cannot completely rule out contamination bias, but it is unlikely that SMS reminders would have been forwarded from intervention to control hospitals. However, even if this contamination of SMS existed from intervention to control hospitals, this would result in an underestimation of the effect of the intervention and would result in a conservative estimate of the effect. Finally, because we tested a complex, multifaceted intervention, it was not possible to determine which of its components were primarily responsible for the observed effect.

## Conclusion

The results of this study provide important information to policymakers and other stakeholders in low-resource settings who need to reconcile efforts to increase access to caesarean birth on the one hand without contributing to the rise in non-medically indicated caesareans on the other hand. The intervention could be easily adapted to the varying healthcare systems in these countries to improve the quality of care.

## Additional files

Additional file 1: The relevant references. (DOC 32 kb)

Additional file 2: List of experts. (DOC 26 kb)

Additional file 3: Algorithm vaginal birth after caesarean section. (PDF 138 kb)

Additional file 4: Algorithm pre-eclampsia. (PDF 192 kb)

Additional file 5: Algorithm eclampsia. (PDF 132 kb)

Additional file 6: Algorithm fetal distress. (PDF 90 kb)

Additional file 7: Algorithm prolonged labor. (PDF 90 kb)

Additional file 8: SMS-based reminders. (DOC 32 kb)

Additional file 9: Approval of Research Ethics Committee of University of Montreal. (PDF 44 kb)

Additional file 10: Approval of Burkina Faso Ethic Committee. (JPG 233 kb)

## Abbreviations

CHUL: Hospital Center of Laval University
HRP: Training in Human Reproduction
IRD: Institut de Recherche pour le Développement
IRSPUM: University of Montreal Public Health Research Institute, Montreal, Canada
