# Supplementary material for: DECIDE: a cluster-randomized controlled trial to reduce unnecessary caesarean deliveries in Burkina Faso
Source: BMC Med. 2019 May 2;17:87. doi: 10.1186/s12916-019-1320-y (PMC6498483; doi:10.1186/s12916-019-1320-y)
Supplement: Supplementary file 1 — The relevant references. (DOC 32 kb) [file 12916_2019_1320_MOESM1_ESM.doc]

1) Rongère J, Domecq S, Djihoud A, Pourin C. Accouchements par césarienne

programmée à terme avant début de travail. Revue de pertinence.

Bordeaux: CCECQA; 2013.

2) ACOG Practice Bulletin no. 115: Vaginal birth after previous cesarean

delivery. Obstet Gynecol. 2010;116(2 Pt 1):450–63.

3)Alauddin M, Bal R, Kundu MK, Dey S, Biswas A. Current trends in delivery of

eclampsia patients. J Indian Med Assoc. 2009;107(10):672–4.

4) Collège National des Gynécologues et Obstétriciens Français (CNGOF).

Recommandations pour la pratique clinique. Accouchement en cas d’utérus

cicatriciel. 36èmes Journées Nationales. Paris; 2012.

5) Collège National des Gynécologues et Obstétriciens Français (CNGOF). Item

17, item 218: Principales complications de la grossesse – Pré-éclampsie et

syndrome pré-éclamptique. Université Médicale Virtuelle Francophone;

2010. Available from: http://campus.cerimes.fr/gynecologie-et-obstetrique/

enseignement/item17_5/site/html/. Accessed 8 Sept 2015.

6) Elkousy MA, Sammel M, Stevens E, Peipert JF, Macones G. The effect of birth

weight on vaginal birth after cesarean delivery success rates. Am J Obstet

Gynecol. 2003;188(3):824–30.

7) Esposito MA, Menihan CA, Malee MP. Association of interpregnancy interval

with uterine scar failure in labor: a case-control study. Am J Obstet Gynecol.

2000;183(5):1180–3.

8) Rooth G, Huch A, Huch R. Guidelines for the use of fetal monitoring. Int J

Gynecol Obstet. 1987;25:159–67.

9) Fraser WD, Turcot L, Krauss I, Brisson-Carrol G. WITHDRAWN: Amniotomy for

shortening spontaneous labour. Cochrane Database Syst Rev. 2006;(3):

CD000015. Available from: http://www.ncbi.nlm.nih.gov/pubmed/17636574.

Accessed 8 Sept 2015.

10) Hannah ME, Hannah WJ, Hewson SA, Hodnett ED, Saigal S, Willan AR. Term

Breech Trial Collaborative Group. Planned caesarean section versus planned

vaginal birth for breech presentation at term: a randomised multicentre

trial. Lancet. 2000;356(9239):1375–83.

11) Hofmeyr GJ, Hannah ME. Planned caesarean section for term breech

delivery. Cochrane Database Syst Rev. 2000;(2):CD000166. Available from:

http://www.ncbi.nlm.nih.gov/pubmed/10796165. Accessed 8 Sept 2015.

12) Johanson R, Menon V. WITHDRAWN: Vacuum extraction versus forceps for assisted

vaginal delivery. Cochrane Database Syst Rev. 2010;(11):CD000224. Available from:

http://www.ncbi.nlm.nih.gov/pubmed/21069665. Accessed 8 Sept 2015.

13) Liston R, Sawchuck D, Young D. Fetal health surveillance: antepartum and

intrapartum consensus guideline. J Obstet Gynaecol Can. 2007;29(9 Suppl 4):S3–56.

14) Marpeau L. Traité d’obstétrique. Paris: Elsevier Masson; 2010.

15) Martel MJ, MacKinnon CJ, et al. Guidelines for vaginal birth after previous

caesarean birth [Internet]. February 2005, pp. 164–74. Report No. 155.

Available from: http://sogc.org/wp-content/uploads/2013/01/155E-CPGFebruary2005.

pdf. Accessed 8 Sept 2015.

16) Mashiloane CD, Moodley J. Induction or caesarean section for preterm

pre-eclampsia? J Obstet Gynaecol. 2002;22(4):353–6.

17) Rouse DJ, Owen J, Savage KG, Hauth JC. Active phase labor arrest: revisiting

the 2-hour minimum. Obstet Gynecol. 2001;98(4):550–4.

18) Royal College of Obstetricians & Gynaecologists. Operative vaginal delivery

(Green-top guideline No. 26). London: RCOG; 2012. Available from: https://

www.rcog.org.uk/en/guidelines-research-services/guidelines/gtg26/.

Accessed 8 Sept 2015.

19) Thomas J, Callwood A, Paranjothy S. National Sentinel Caesarean Section

Audit: update. Pract Midwife. 2000;3(11):20.

20) Vayssière C, Beucher G, Dupuis O, Feraud O, Simon-Toulza C, Sentilhes L, et al.

Instrumental delivery: clinical practice guidelines from the French College of

Gynaecologists and Obstetricians. Eur J Obstet Gynecol Reprod Biol. 2011;

159(1):43–8.

21) Cargill YM, MacKinnon CJ, Arsenault M-Y, Bartellas E, Daniels S, Gleason T, et al.

Guidelines for operative vaginal birth. J Obstet Gynaecol Can. 2004;26(8):747–61.

22) Multidisciplinary management of severe pre-eclampsia (PE). Ann Fr Anesth

Reanim. 2009;28(3):275–81.

23) SOGC clinical practice guidelines. Guidelines for vaginal birth after previous

caesarean birth. Number 155 (Replaces guideline Number 147), February

2005. Int J Gynaecol Obstet. 2005;89(3):319–31.

24) Hill JB, Ammons A, Chauhan SP. Vaginal birth after cesarean delivery:

comparison of ACOG practice bulletin with other national guidelines. Clin

Obstet Gynecol. 2012;55(4):969–77.
