# Supplementary figures and images for: DECIDE: a cluster-randomized controlled trial to reduce unnecessary caesarean deliveries in Burkina Faso
Source: BMC Med. 2019 May 2;17:87. doi: 10.1186/s12916-019-1320-y (PMC6498483; doi:10.1186/s12916-019-1320-y)

## Algorithm: Vaginal birth after caesarean (VBAC)

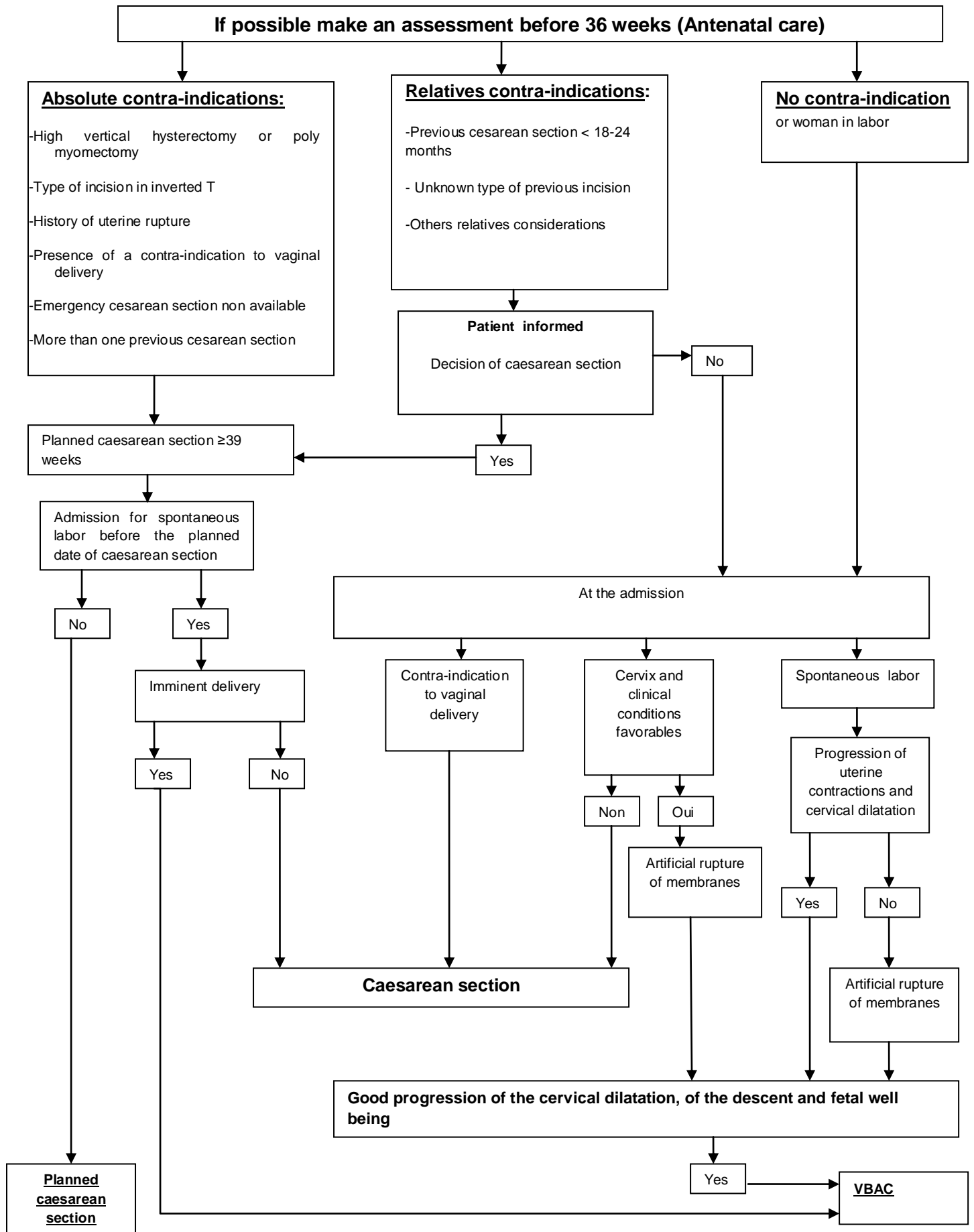

Supplement: Supplementary file 3 — Algorithm vaginal birth after caesarean section. (PDF 138 kb) [file 12916_2019_1320_MOESM3_ESM.pdf]

## Algorithm: Fetal distress during labor

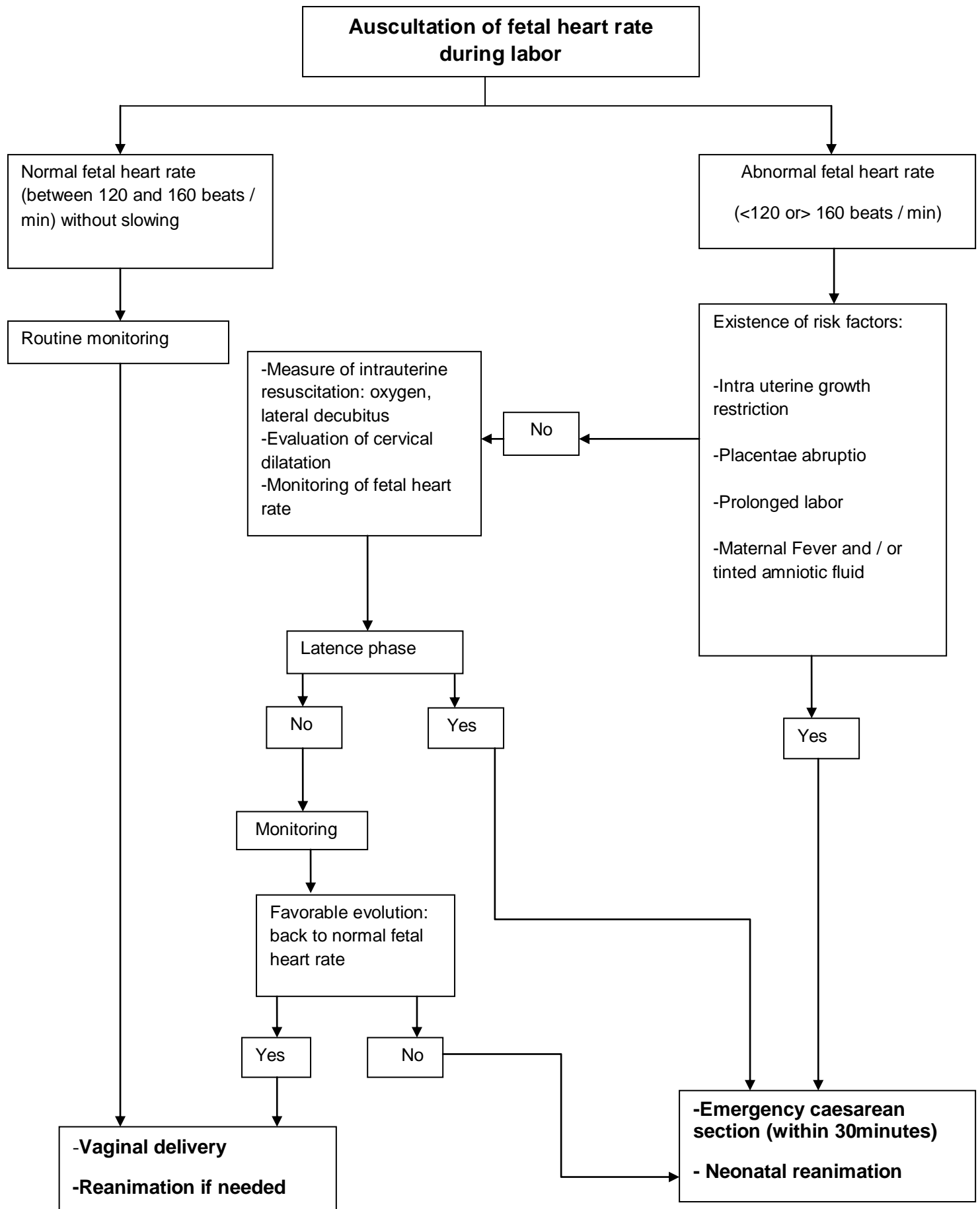

Supplement: Supplementary file 6 — Algorithm fetal distress. (PDF 90 kb) [file 12916_2019_1320_MOESM6_ESM.pdf]

## Algorithm : Prolonged labor

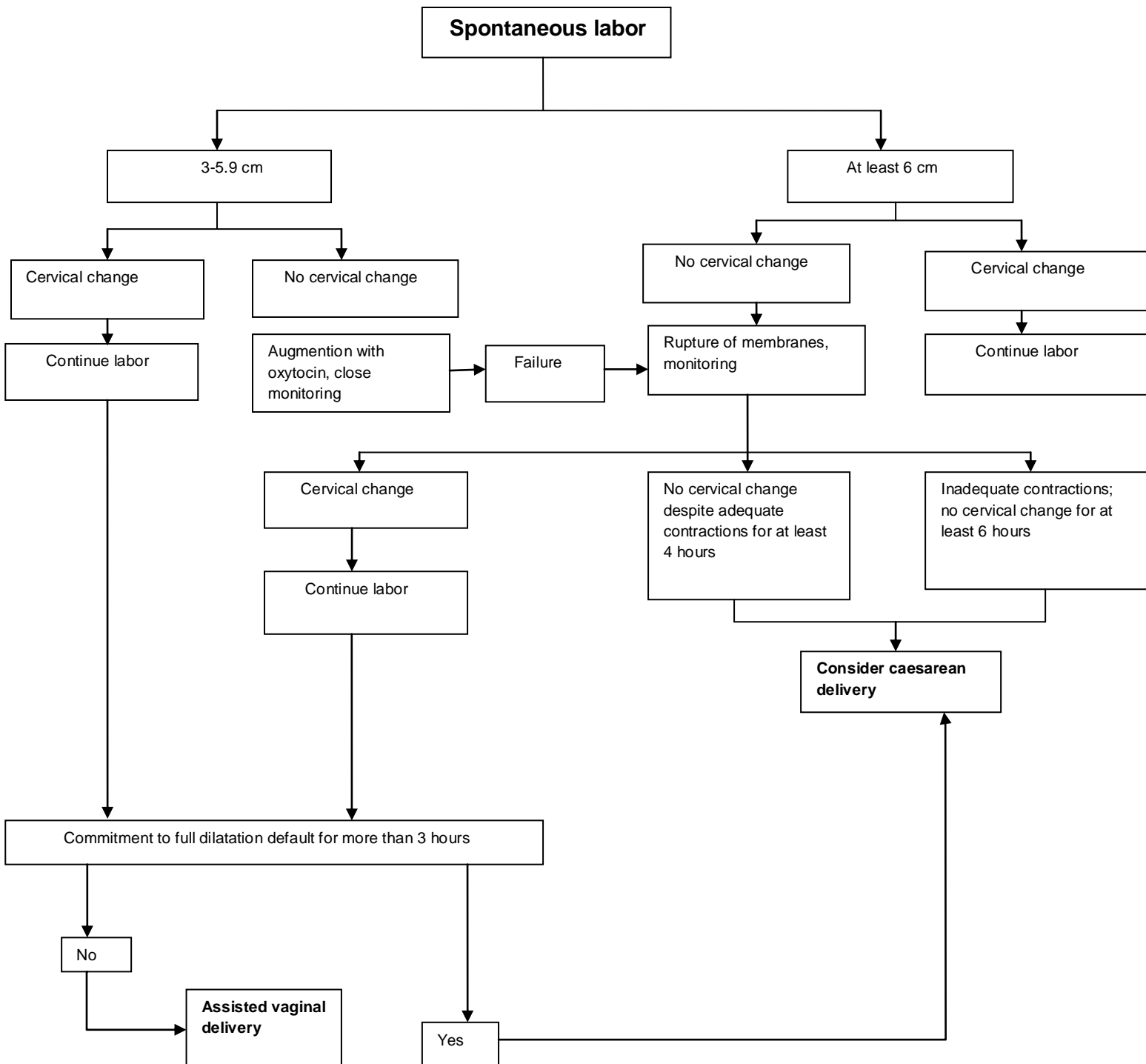

Supplement: Supplementary file 7 — Algorithm prolonged labor. (PDF 90 kb) [file 12916_2019_1320_MOESM7_ESM.pdf]

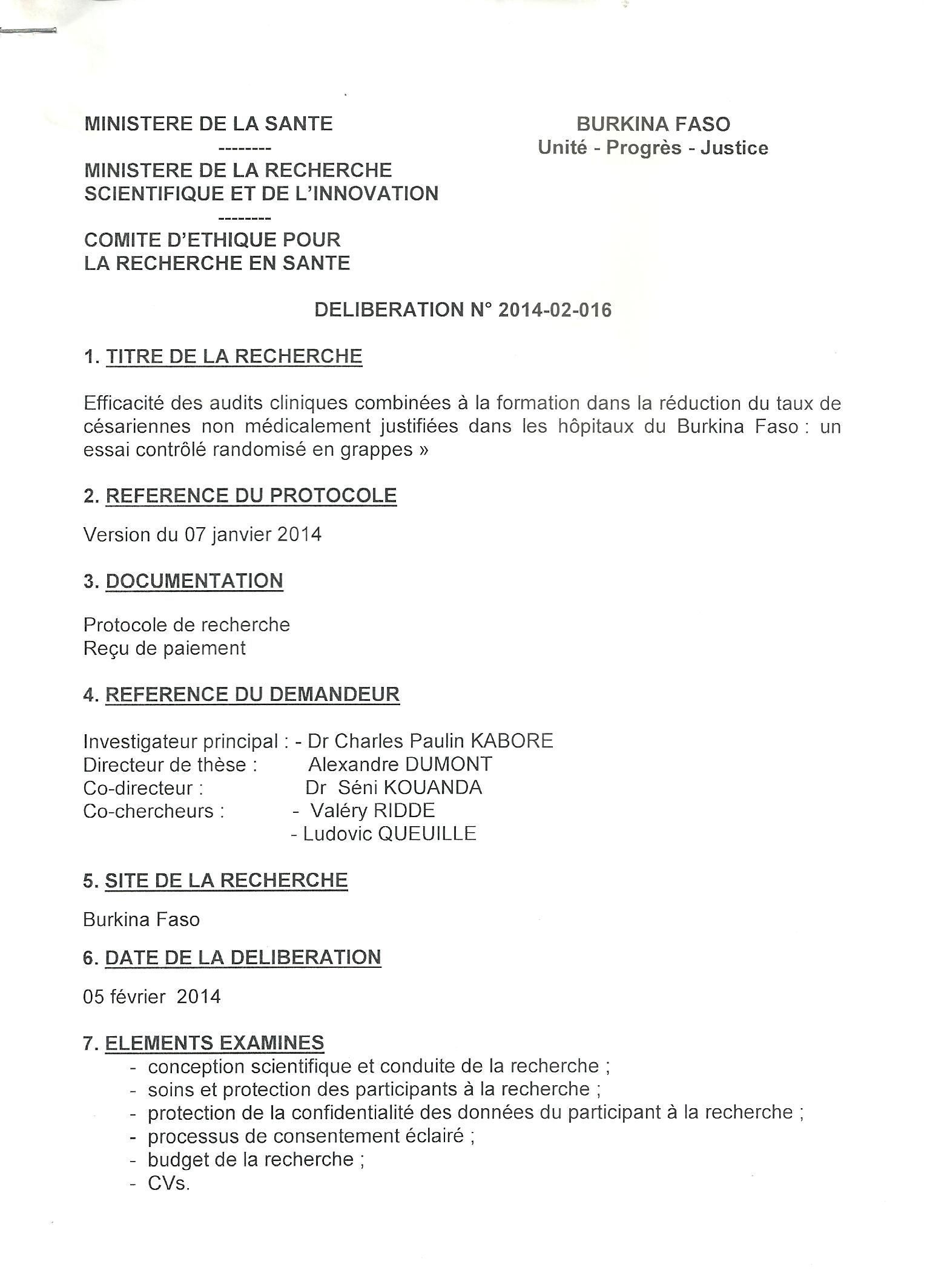

Supplement: Supplementary file 10 — Approval of Burkina Faso Ethic Committee. (JPG 233 kb) [file 12916_2019_1320_MOESM10_ESM.jpg]
