# Supplementary material for: DECIDE: a cluster-randomized controlled trial to reduce unnecessary caesarean deliveries in Burkina Faso
Source: BMC Med. 2019 May 2;17:87. doi: 10.1186/s12916-019-1320-y (PMC6498483; doi:10.1186/s12916-019-1320-y)
Supplement: Supplementary file 8 — SMS-based reminders. (DOC 32 kb) [file 12916_2019_1320_MOESM8_ESM.doc]

**CLINICAL RECOMMENDATIONS

General recommendation**

Caesarean section should not be indicated immediately in a woman with no cons-indication for vaginal delivery.

**Caesarean section for history of cesareans**

1. Any woman with a history of one segmental transverse cesarean without cons-indication for vaginal delivery, with a single fetus pregnancy with cephalic presentation should benefit from a trial of labor in a center where emergency caesarean section is available.

2. In the event of imminent delivery in women with history of cesareans, we must promote vaginal birth.

3. An iterative caesarean section should not be indicated for short reproductive inter-space if the last Caesarean section was completed within less than 24 months.

4. If the trial of labor is indicated for a woman with history of cesareans and if the center where you work cannot perform an emergency C-section, you have to refer this woman before the onset of labor to another center where this is possible.

**Caesarean section for dystocia / prolonged labor**

1. Caesarean section should not be indicated for prolonged or obstructed labor in the presence of stagnant dilation less than 6 hours for a cervical dilatation less than 6 cm.

2. The C-section for prolonged or obstructed should not be indicated in case of stagnation of cervical dilatation less than 3 hours for a cervical dilatation more than 6 cm.

3. Caesarean section for prolonged or obstructed labor should not be indicated for stagnation of cervical dilation without started adequate treatment.

4. Caesarean section for failure of commitment should not be indicated before three hours of full dilation monitoring.

5. Any primipare woman with clinically contracted pelvis and a single fetus in cephalic presentation should benefit from a trial of labor in a center where an emergency caesarean section can be performed.

**Caesarean section for eclampsia / preeclampsia**

1. A caesarean should not be indicated before labor for preeclampsia if the blood pressure is stable and controlled under medical treatment and in the absence of signs of fetal distress.

2. Caesarean section for preeclampsia should not be indicated during labor in case of fetal death or imminent delivery or if no immediate signs of severity.

3. In case of imminent delivery in a woman having eclampsia crises we must promote assisted vaginal delivery by vacuum.

**Caesarean section for fetal distress**

1. Caesarean section should not be indicated during labor for fetal distress in the absence of abnormal fetal heart rate or if the amniotic fluid is clear

2. In case of an abnormal fetal heart rate in active labor stage, caesarean section should be indicated after failure of intrauterine resuscitation.

3. In case of fetal distress at fully dilatation, the instrumental delivery should be preferred.
