# Supplementary material for: DECIDE: a cluster-randomized controlled trial to reduce unnecessary caesarean deliveries in Burkina Faso
Source: BMC Med. 2019 May 2;17:87. doi: 10.1186/s12916-019-1320-y (PMC6498483; doi:10.1186/s12916-019-1320-y)
Supplement: Supplementary file 9 — Approval of Research Ethics Committee of University of Montreal. (PDF 44 kb) [file 12916_2019_1320_MOESM9_ESM.pdf]

CHUM
Comité d'éthique de la recherche du CHUM
Tour Viger, 900 rue St-Denis, 3e étage
Montréal (Québec) H2X 0A9

Le 26 mars 2014

Docteur Alexandre Dumont
Axe de recherche : santé mondiale

a/s : Isabelle Agier
courriel : coordinationvr@gmail.com

Objet :	13.356 – Approbation initiale et FINALE CÉR restreint
	**Efficacité des audits cliniques combinés à la formation dnas la réduction du taux de césarienne non médicalement justifiées dans les hôpitaux du Burkina Faso: un essai contrôlé randomisé en grappes**

Docteur Dumont,

Nous accusons réception, en date du 18 mars 2014, des documents soumis en vue de l'approbation du projet mentionné en rubrique. Votre projet a été évalué en comité restreint compte tenu qu'il s'agit d'un projet considéré à risque minimal pour les participants. Nous avons examiné les documents suivants :

- Formulaire de demande d'évaluation éthique d'un projet (formulaire 11)
- Protocole de recherche (Cesarienne-EfficacitéAudit&Formation_ProtocoleSoumis-20Jan14-1.doc), version 1, daté du 7 mars 2014
- Formulaire d'information et de consentement, version 1, daté du 7 mars 2014
- Formulaire A, version 1, daté du 18 mars 2014 .pdf)
- Budget, version 1, daté du 7 mars 2014
- Engagement du chercheur, daté du 18 mars 2014
- Rapport (Approbation CE Burkina Faso)

Le tout étant jugé satisfaisant, il nous fait plaisir de vous informer que la présente constitue l'approbation finale de votre projet, **valide pour un an à compter du 26 mars 2014**. Vous devrez compléter le formulaire de renouvellement que nous vous ferons parvenir annuellement. De même, vous devrez soumettre pour approbation préalable, toute demande de modification ou document de suivi requis par le comité d'éthique conformément à ses Statuts et Règlements et ce via Nagano.

Le comité suit les règles de constitution et de fonctionnement de l'Énoncé de Politique des trois Conseils (ÉPTC 2) et des Bonnes pratiques cliniques de la CIH.

Pour toute question relative à cette correspondance, veuillez communiquer avec la personne soussignée via NAGANO, ou avec sa collaboratrice, par téléphone ou courriel : ghislaine.otis.chum@ssss.gouv.qc.ca – **514 890-8000 poste 14485.**

Vous souhaitant la meilleure des chances dans la poursuite de vos travaux, nous vous prions d'accepter, cher Docteur, nos salutations distinguées.

Camille Assemat
Vice-présidente
Comité d'éthique de la recherche du CHUM
